# Intraoperative Functional Ultrasound Imaging of Human Brain Activity

**DOI:** 10.1038/s41598-017-06474-8

**Published:** 2017-08-04

**Authors:** Marion Imbault, Dorian Chauvet, Jean-Luc Gennisson, Laurent Capelle, Mickael Tanter

**Affiliations:** 1 Institut Langevin – Ondes et Images, ESPCI ParisTech, PSL Research University, CNRS UMR 7587, INSERM U979, 17 rue Moreau, 75012 Paris, France; 2 Service de neurochirurgie, Groupe Hospitalier Pitié-Salpêtrière, Bâtiment Babinski 47-83 Boulevard de l’Hôpital, 75013 Paris, France; 3 Service de neurochirurgie, Fondation Rothschild, 29 rue Manin, 75019 Paris, France

## Abstract

The functional mapping of brain activity is essential to perform optimal glioma surgery and to minimize the risk of postoperative deficits. We introduce a new, portable neuroimaging modality of the human brain based on functional ultrasound (fUS) for deep functional cortical mapping. Using plane-wave transmissions at an ultrafast frame rate (1 kHz), fUS is performed during surgery to measure transient changes in cerebral blood volume with a high spatiotemporal resolution (250 µm, 1 ms). fUS identifies, maps and differentiates regions of brain activation during task-evoked cortical responses within the depth of a sulcus in both awake and anaesthetized patients.

## Introduction

Various methods are used to image brain activity *in vivo*. It can be directly detected by neuronal electrical activity imaging (electrocortical stimulation mapping^1^, calcium imaging^2^, voltage sensitive dyes^3^) or indirectly by imaging the haemodynamic changes induced by the neurovascular coupling in the vessels surrounding the activated neurons (multiphoton microscopy^4^, optical coherence tomography^5^, positron emission tomography (PET)^6^ and functional magnetic resonance imaging (fMRI)^7, 8^).

Although functional neuro-imaging during surgery would be highly beneficial, none of these techniques provides a simple and portable intraoperative brain imaging modality within the depth of a sulcus. The goal of intraoperative functional mapping is to maximize tumour removal while preserving functional brain areas, thus minimizing the risk for postoperative deficits and improving long-term survival^9^. Ultrasound can potentially address this need. Indeed, ultrasound imaging achieves good, in-depth spatiotemporal resolution and is used intraoperatively to localize tumour tissue. However, the use of Doppler ultrasound has been limited to the imaging of major vessels due to its poor sensitivity. To overcome this limitation, functional ultrasound (fUS) was developed^10^. This technique allows for high spatiotemporal resolution imaging (250 µm, 1 ms) of brain microvasculature dynamics in response to brain activation without the need for a contrast agent.

This fUS method relies on a new, ultrasensitive power Doppler imaging sequence that is sensitive enough to detect blood flow in most cerebral vessels (down to ~1 mm.s^−1^ blood flow speed). The repeated acquisition of such ultrasensitive Doppler images over time allows for the visualization of flow dynamics in the vessels that are modulated by local neuronal activity. This new sequence is derived from the key concept of ultrafast imaging^11^, which is based on the emission of very high frame rate ultrasonic plane waves (~20 kHz). To attain ultrasensitive Doppler information, this approach must be combined with an accumulation step. This step involves exploiting hundreds of time samples acquired at ultrafast frame rates during periods that are shorter than the typical observation times for brain haemodynamics (typically 1s). Applied to the rat brain, fUS imaging was shown to be able to map brain activation at a high spatiotemporal resolution with a high signal-to-noise ratio (SNR)^12^. Here, we adapted and implemented this new modality intraoperatively in humans.

## Results

### The cohort of patients

This study provides a proof of concept that ultrasound can map brain activation in humans based on our experience with 33 adults (19 women, 14 men, aged 24–64 years, mean 42 years). All patients provided informed consent and ethical considerations were previously validated by our institutional ethics committee, “Comité de Protection des Personnes – Ile-de-France VI – Pitié Salpêtrière” (CPPIDF6, CPP n° 72–15). All patients had low-grade gliomas and were included because intraoperative functional mapping was planned in the removal of their tumours.

### The gold standard for direct intraoperative functional mapping

Low-grade gliomas are brain tumours that often extend into highly functional areas and modify the usual cortical functional anatomy. Therefore, intraoperative electrocortical stimulation mapping (ESM) is commonly used to assess functional cortical reorganisation^13^. In a meta-analysis of 8,091 patients^14^, much better neurological outcomes were observed with the use of ESM and the gross total resections were achieved more frequently with ESM (74.9%) than without ISM (58.1%). Although ESM presents some risks, especially intraoperative seizures, it is the current gold standard for intraoperative superficial functional cortical mapping^15, 16^. We used this technique as a reference in our study.

### Functional areas studied based on the neurovascular coupling

We used fUS (Methods, Ultrasonic imaging, Ultrasonic signal processing) to image task-evoked brain activation during tumour surgery after opening the skull and the dura mater. fUS determines regions of brain activity based on increased cerebral blood volume due to neurovascular coupling. A typical haemodynamic response to cortical neuronal activation in adults is increased blood flow^17^. Blood constants quantification can subsequently provide an indirect picture of neuronal activity. Since fUS efficiency relies on an intact auto-regulatory response similar to fMRI, some pathological states can affect this process, such as type 2 diabetes^18^, and will require vigilant investigation.

In five patients, surgical complications prevented us from performing fUS during surgery (Methods, Surgery). These five patients were excluded from further analysis. 27 patients were awake and under local anaesthesia during ESM and 6 patients were under general anaesthesia (Methods, Standards for Intraoperative Cortical Localization). Among the 28 patients who were able to undergo fUS, 46 tasks were tested (a mean of 2 tested areas per patient). In three cases, remodelling due to tumour growth limited access to a single functional area using electric stimulation at the extreme border of the trepanation window (just under the bone). This location could be reached using the electrical stimulator ball tip probe, but it was not accessible with our ultrasound probe. Since the probe could not be placed on the functional area, but rather only a few millimetres away from the bone window, functional ultrasound was not able to detect the stimulus as expected. These three cases were excluded from the statistical analysis.

fUS succeeded in the detection and mapping of the corresponding functional areas in 43 cases (100%) when the ultrasound probe could be placed on the functional area. Depending on lesion location, we were able to localize functional areas that correspond to 16 different tasks, including tasks that involve the hand, index finger, thumb, wrist, elbow and mouth for both motor and sensory tasks (Supplementary Fig. 1).

### Deep cortex activation maps

**Figure 1:**
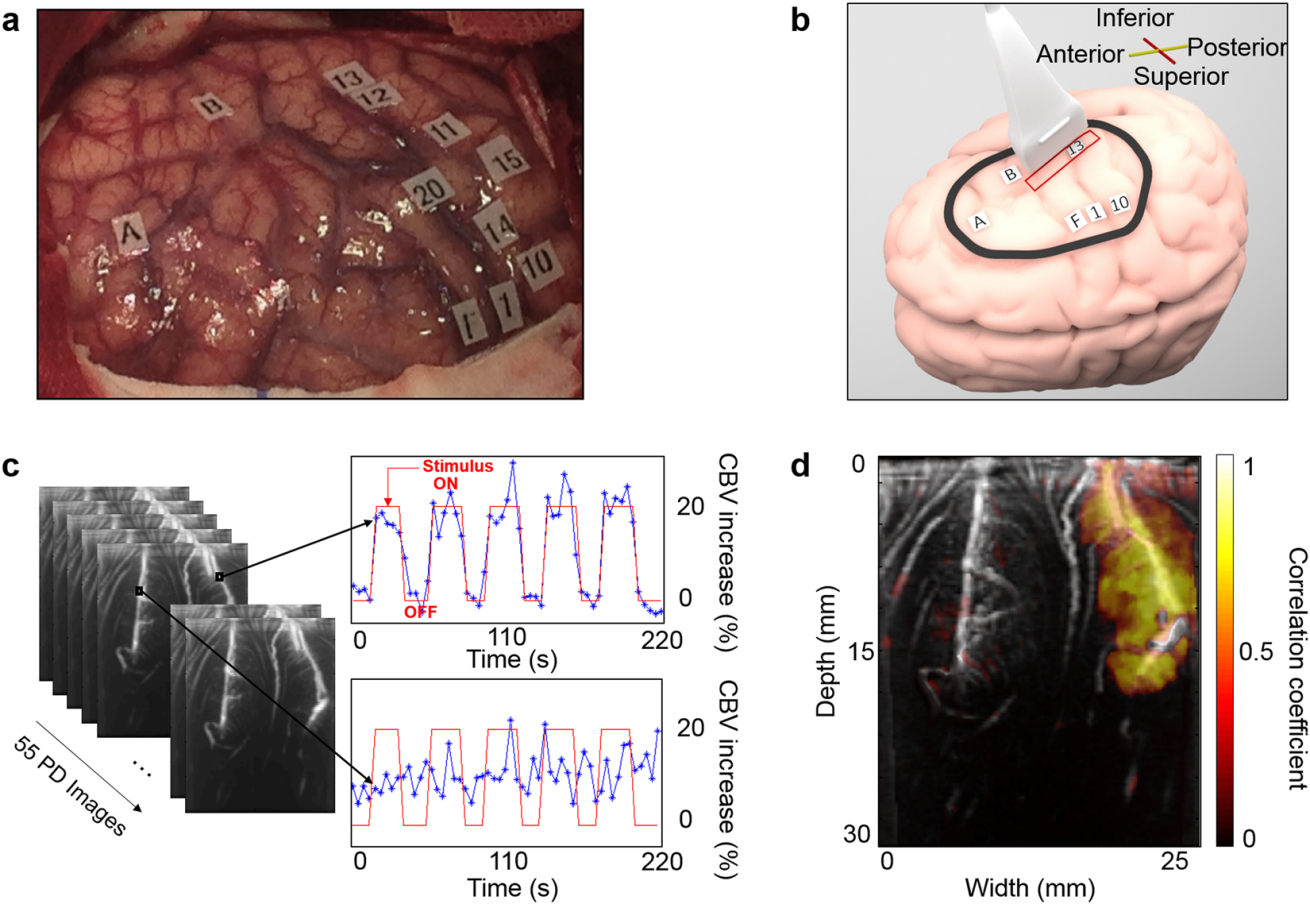
Intraoperative fUS imaging of “mouth sensitive”-evoked brain activation in one patient. (**a**) Superficial ESM activation map; the letters show the tumour position, numbers from 1 to 10 represent the motor cortex and numbers from 11 to 20 represent the somatosensory cortex. (**b**) The probe position on the cortex (red rectangle) on the “mouth sensory” ESM tag 13. The drawing of the brain was created by Alexandre Dizeux. (**c**) Power Doppler fUS images are acquired every 3 s when the awake patient is performing the corresponding task (“mouth opening and closing”). This motor task was chosen to stimulate a sensitive area because the patient’s lips touching each other is a strong sensitive stimulation. The task pattern (red line) consisted of six blocks of reference conditions (OFF) and five blocks of tasks (ON). The cerebral blood volume (CBV) increase is plotted in a percentage relative to the baseline CBV. Variations in blood volume during ultrasound acquisitions are presented in Supplementary Video 1 for this task and this patient. (**d**) The in-depth activation map obtained for the task “mouth opening and closing” when the probe is on the “mouth sensory” ESM tag 13.

The precise location of the task-related areas at the cortical surface was first determined by ESM. Then, the ultrasound probe was placed on one chosen ESM tag during a task-related stimulus (e.g., Fig. 1a,b). fUS acquisitions were performed during one heart cycle (1s) at a 1 kHz frame rate and were repeated every 3 s (Fig. 1c). Stimulation induced a steady increase in blood volume in the associated cortical area, with a 20% increase in the cerebral blood volume (CBV) compared to baseline (Fig. 1c and supplementary video 1). Maps of the activated pixels were built that show the correlation coefficient *r* between the power Doppler signal and the stimulus temporal pattern. Activation maps (Fig. 1d) showed a significant and localized correlation (*r *> 0.41, Methods, Activation maps) between the power Doppler (PD) signal and the task pattern during the task.

### fUS ability to differentiate two neighbouring functional areas

**Figure 2:**
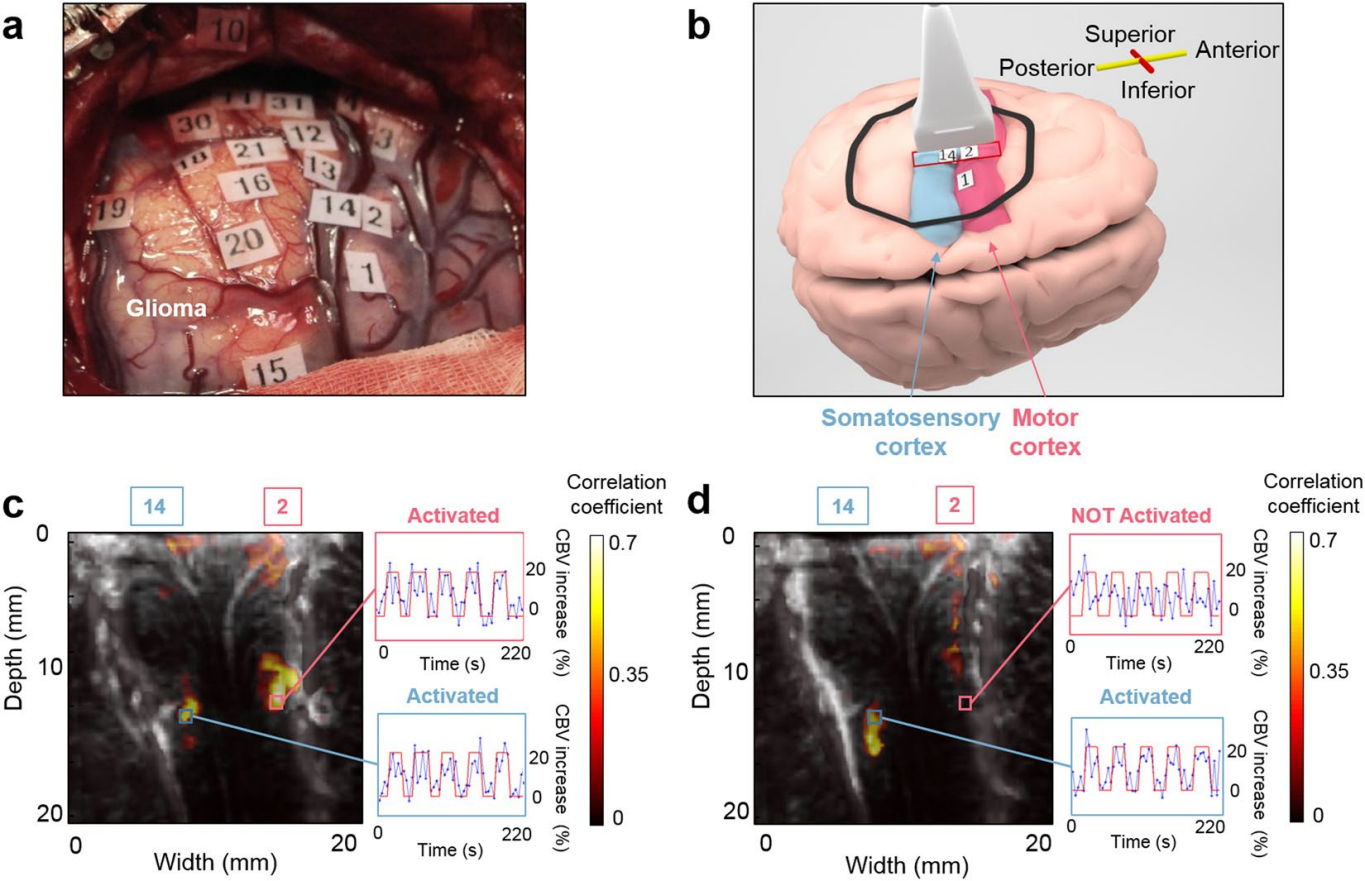
Comparison between the intraoperative functional imaging of the motor and the somatosensory cortices. (**a**) Superficial ESM activation map. Numbers from 1 to 10 represent the motor cortex and numbers from 11 to 20 represent the somatosensory cortex. (**b**) The probe position on the brain cortex (red rectangle). The probe is across the “2–5 fingers motor” (ESM tag 2) and the “4–5 fingers sensory” (ESM tag 14) for both activation maps presented in (**c**) and (**d**). The drawing of the brain was created by Alexandre Dizeux. (**c**) The in-depth activation map obtained for the task “2–5 fingers motor”. The correlation map illustrates that both the motor (ESM tag 2) and the somatosensory (ESM tag 14) cortices are implicated in the task. (**d**) The in-depth activation map obtained for the task “4–5 fingers sensory”. The correlation map illustrates that only the somatosensory cortex (ESM tag 14) is implicated in this task. In (**c**) and (**d**), the cerebral blood volume (CBV) increase is plotted in a percentage relative to the baseline CBV for different regions of interest.

To verify that fUS imaging enables the precise localization of functional areas, we positioned the probe across two functional areas (Fig. 2a,b). For a motor task, the in-depth activation maps reveal that both the motor and the somatosensory cortex are implicated in the task (Fig. 2c). For a sensory task, only the somatosensory cortex is implicated in the task (Fig. 2d). A tiny activation barely above the noise level can be seen in the small areas of the upper region of the motor cortex. This tiny activation detected in the upper cortex motor zone during the sensitive task (Fig. 2d) could potentially be explained by a small motion by the finger during the stimulus as the caress was intense.

### Control tasks

As a control test for two different tasks, we used fUS imaging to verify that no correlation (*r*_*correlation*_ ≫ 0.41) between the Doppler signal and the contralateral task pattern was found in the brain region under the ESM tag that corresponds to the initial task (spatial average *r*_*mean*_ = 0.078 ± 0.130).

## Discussion

The determination of individual functional cortical mapping is essential for the optimal performance of surgery in highly functional areas, minimizing the risk of postoperative deficits. Although the accuracy of intraoperative ESM is high, key disadvantages can present challenges, such as prolonged operative times and seizure risk. As an example, in our study, ESM induced epileptic seizures in 5 cases, which were quickly stopped medically with a cold physiologic serum injection. ESM can be stressful for a patient and cannot be performed in some cases due to clinical considerations. Additionally, neurosurgeons do not normally stimulate within the depth of a non-opened sulcus^19^ and therefore do not stimulate the whole cortex in depth. For the fMRI activation maps, the technique provides non-invasive functional mapping for the surgeon to plan the lesion resection. However, brain shifts after dural opening remain a major issue when using MRI neuronavigation and the correlation between fMRI activation sites and positive ESM sites is not always high.

In this context, the ability of ultrafast ultrasound to intraoperatively map the brain with high SNR stimulus-based neuronal activations in the deep cortex during brain surgery is of great interest. We developed ultrafast Doppler sequences and data processing for fUS imaging adapted to intraoperative functional imaging in humans. fUS imaging with a 500 Hz frame rate was able to determine regions of brain activity based on cerebral blood volume increases during a task due to neurovascular coupling. The neurovascular coupling assessment is not restricted to pixels where the vessels are visible in the Power Doppler image. Indeed, a difference exists between the detection of Doppler signal changes in small vessels (neurofunctional ultrasound) and the imaging of vessels (ultrasound angiography). In clinical fUS imaging, we can follow changes in blood flow at very low blood flow speeds (3 to 10 mm/s) that correspond to arterioles and venules that are smaller than the pixel size (250 µm) and are therefore non-visible on the Power Doppler image.

Since glioma surgery requires a large bony window, the ultrasound mapping procedure is feasible in many cases. The exploration depth in this proof of concept study was limited to 4 cm due to the features of the ultrasound probe (6 MHz central frequency, 30 mm elevation depth). Changing to a lower transmission frequency and adapting the probe’s geometry would permit the imaging of deeper regions.

Our technique is currently limited in 2D, but high-resolution ultrasound 3D images of the brain vasculature will be possible in the very near future. 4D vascular imaging was recently performed in rodents using a tomographic approach^20^. However, this approach was not performed in real time because it only allows the acquisition of one 2D plane per cardiac cycle and the acquisitions have to be repeated plane by plane. However, the challenge in achieving such 3D images of the brain vasculature after opening the dura within a single cardiac cycle is only technological and relies on the development of ultrasonic probes and scanners that perform 3D ultrafast Doppler imaging. 3D ultrasound images are feasible using either matrix transducer arrays that consist of thousands of piezo-elements driven by ultrasound research scanners^21^ or using a new type of transducer technology such as Row Column Transducer Arrays^22–24^.

We tested the fUS technique on both awake and anaesthetized patients. In both cases, fUS was able to detect the activation of functional areas in response to stimulation with a 20% increase in cerebral blood volume compared to baseline. Sixteen different stimuli were tested, and when the ultrasound probe was positioned on one ESM tag, fUS always succeeded in functional area localization. We also performed control tests to verify that no correlation existed between the Doppler signal and the task pattern in the case of a contralateral stimulus. Finally, we demonstrated that intraoperative fUS was able to differentiate two neighbouring functional areas when the ultrasound probe was placed across two different ESM tags.

We demonstrated that fUS is able to identify, map and differentiate regions of brain activation in two dimensions during task-evoked cortical responses within the depth of a sulcus, in both awake and anaesthetized patients.

## Methods

### Ethical Approval

The clinical investigation (N° ID-RCB 2015 A00661-48) was accepted by French regulatory agencies (ANSM, reference B150800-31) and by the Comité de Protection des Personnes CPP Ile de France VI (CPP n° 72–15). All patients gave their informed consent. All ultrasound experiments were performed in accordance with this ethics committee.

### Surgery

Local anaesthesia with intravenous sedation (propofol, remifentanil) was chosen for 27 patients undergoing surgery with intraoperative electrocortical simulation mapping (ESM). In these cases, local anaesthesia was performed only during cranial flap opening and closing and the patients did not receive any sedative during the ultrasound acquisition. Six more patients had surgery under general anaesthesia (propofol, sufentanil, sevofluran), and ESM was performed while they were unconscious. Before imaging the patient, craniotomy and durotomy were performed on the appropriate site to proceed with the lesion resection.

In five patients, surgical complications prevented the performance of fUS during surgery: prolonged seizure at the very beginning of ESM, dramatic difficulties in talking and thinking by the patient (issues with talking was the only ESM tag), very intense venous haemorrhage at the slightest electrocortical stimulation (ESM was not possible), and one patient did not wake up during the surgery and haematoma formation dissuaded the use of ESM, so no functional area was detected by ESM.

### Standards for Intraoperative Cortical Localization

Bipolar cortical stimulation was performed with an electrical stimulator (ball tip probe, Ball tip Ø = 2.0 mm, 1–15 mA; inomed Medizintechnik GmbH, Germany). 4.5 to 15 mA was necessary to induce a response in the patients under general anaesthesia, and 1.5 to 5.5 mA was necessary for the patients under local anaesthesia. Each stimulation train was limited to two to four seconds. Motor responses, speech arrest, particular sensations and experiences were documented. The awake patient was able to communicate and perform tasks that depended on the location of the lesion and on the nearby functional cortex. In the patients under general anaesthesia, motor responses were observed by a third person. Based on the results, tags were placed over each stimulation site (the central point between the electrode tips).

An average of two cortex simulations were performed on each patient. Motor cortex localization was performed with the elicitation of a movement in response to electrocortical stimulation. Sensory cortex localization was ascertained by localized paresthesias reported by the patients in response to primary somatosensory cortex stimulation. Localization of the language/speech cortex was mostly elicited, given the cortical areas stimulated, by speech arrest and anomia in response to intraoperative cortical stimulation.

### Ultrasonic Imaging

Neuronal activity induces increased blood volumes within small arterioles and venules^25^ and within capillaries^26, 27^. Drew and colleagues demonstrated that, on average, the speed and flux (cells per unit time) of RBCs covaried linearly at low values of flux^25^. Therefore, both the average velocity and density of RBCs are greater at high values of flux than at low values. Using fMRI, Jin and colleagues^28^ also showed that CBF and CBV increased during visual stimulation. The fUS method measures local changes in blood volume and can therefore detect this local increased perfusion during stimulation.

The power Doppler signal is known to be directly proportional to the number of moving red blood cells in the sample volume. In other words, it is proportional to the local cerebral blood volume^29–31^. Importantly, this proportionality is only valid if backscattering properties do no vary relative to time. This assumption is valid when red blood cells’ backscattering properties, such as haematocrit and the shear rate, remain time-invariant^32, 33^. The relationship between the Power Doppler signal acquired in fUS imaging and the CBV was studied in calibrated phantom experiments by Mace and colleagues^12^. Flow rate measurements can be derived easily from the same data using the power velocity integral (PVI) method. Instead of calculating the integral of the Doppler signal intensity *P* over the velocity spectrum (Power Doppler ${\int }^{}P.dv$), one could use the integral of the Doppler Intensity multiplied by the velocity ${\int }^{}P.vdv$ that corresponds to the blood flow rate. In the case of neuroimaging, we preferred to use the Power Doppler integral (proportional to blood volume) because cerebral blood volume is a well-known physical parameter for neuroscientists.

Ultrasound images were acquired with an ultrafast ultrasonic device (Aixplorer®, Supersonic Imagine, Aix-en-Provence, France) driving a linear array (SL10-2, Supersonic Imagine, Aix-en-Provence, France, 6 MHz central frequency, 192 elements). One to three sets of fUS images were acquired for every patient and one set included blocks of rest and task periods. One ultrasound acquisition lasts 4 seconds: one second for acquisition (one heart cycle) and three seconds for transfer and recording.

The subjects performed tasks in a block design paradigm, alternating six blocks of reference conditions and five blocks of tasks, with each block lasting 20 s. A full trial session for one patient, with a mean of 2 different tasks, lasts approximately 8 minutes in total.

ESM data were used for ultrasound probe positioning on a targeted functional area. The ultrasound probe was placed in a sterile sleeve filled with sterile ultrasound gel, and the sleeve was placed directly in contact with the cortex after skull and dural opening without any additional coupling liquid. To guarantee its immobility, the ultrasound probe was fixed to a custom autoclavable stainless steel articulated arm. CBV baseline maps were found to be very stable due to this fixed articulated arm setup. The patients were then asked to perform the specific task that corresponded to the ESM tag in the case of a local anaesthesia. In the case of general anaesthesia, a third person was asked to move or stroke the limb segment of interest.

### Ultrasonic signal processing

One dedicated Ultrafast ultrasound sequence that relied on compound plane-wave imaging^11^ was used to image the adult brain during surgery. This sequence attempted to optimize the trade-off between the frame rate and resolution. We intended to perform functional imaging, so we were interested in imaging small vessels where the blood flow is less than 5 cm/s and a frame rate of 500 Hz was enough to correctly sample the data. Consequently, with this frame rate value imposed by the blood flow itself, we chose 6-angle (−3°, −3°, 0°, 0°, +3°, +3°) compound imaging at a firing frequency of 3,000 Hz for slow blood flows. The ultrasound sequence was calibrated with the probe on a certified calibration setup and the acoustic parameters were far below the acoustic intensity limits recommended by the Food and Drug Administration (FDA guidance 510k-Track 3). The acoustic parameters of this ultrasound sequence were as follows: MI = 0.67 (<1.9), ISPTA = 180 mW/cm^2^ (<720 mW/cm^2^) and TIS = 2.4 (<6).

Matlab software (MathWorks) was used to process the signals. For each compounded frame, a set of tilted plane waves^11^ was emitted. For each tilted plane wave emitted, RF data were collected by the transducer and a beam formed as an in-phase/quadrature (IQ) image. These complex images were coherently summed together to generate a compound frame. This operation was repeated at a frame rate of 500 Hz over 1 second to obtain a stack of ultrafast compound frames.

To distinguish the moving red blood cells from the brain parenchyma, we implemented a spatiotemporal filter based on the singular value decomposition of the images stack^34–36^. SVD applied on the IQ data allowed us to take advantage of plane wave transmission and cancel all spatially coherent motion artefacts (vessel pulsatility and vibrations) for optimized CBV mapping. Very slow global motion during long acquisition times was corrected using basic in-plane motion filter processing. After the summation of the intensity of all Doppler frames, a final, very high-quality Ultrafast Doppler image was obtained.

### Activation maps

For the visualization of an activation map, the correlation map was colour-coded and superimposed on the ultrasensitive Doppler image. Activation maps are maps of the Pearson’s product-moment correlation coefficient r between the temporal pattern of the stimulus and the Power Doppler signal for each pixel.

For five cycles, the time course is composed of 55 independent time points. We calculated scores by applying a Fischer’s transform and the level of significance was fixed as z > 3.1 (p < 0.001, one tailed test), which corresponds to r > 0.41. This threshold value was used for all of the activation maps presented in the article.

### Data Availability

The datasets generated and analysed during the current study are available from the corresponding author on reasonable request.

## Electronic supplementary material

Supplementary materials

Supplementary video 1
