# Supplementary material for: Intraoperative Functional Ultrasound Imaging of Human Brain Activity
Source: Sci Rep. 2017 Aug 4;7:7304. doi: 10.1038/s41598-017-06474-8 (PMC5544759; doi:10.1038/s41598-017-06474-8)
Supplement: Supplementary file 1 — Supplementary materials [file 41598_2017_6474_MOESM1_ESM.pdf]

Intraoperative Functional Ultrasound Imaging of Human Brain Activity

Marion Imbault[1x*], Dorian Chauvet[2,3x], Jean-Luc Gennisson[1], Laurent Capelle[2+], Mickael Tanter[1+]

[1] Institut Langevin – Ondes et Images, ESPCI ParisTech, PSL Research University, CNRS UMR 7587, INSERM U979, 17 rue Moreau, 75012 Paris, France
[2] Service de neurochirurgie, Groupe Hospitalier Pitié-Salpêtrière, Bâtiment Babinski 47-83 Boulevard de l'Hôpital, 75013 Paris, France
[3] Service de neurochirurgie, Fondation Rothschild, 29 rue Manin - 75019 Paris, France

[x] Marion Imbault and Dorian Chauvet contributed equally to this work.
[+] Laurent Capelle and Mickael Tanter jointly supervised this work.
* Correspondence to marion.imbault@espci.fr

SUPPLEMENTARY MATERIALS

			Correlation	
	Tasks	Number	**Mean correlation in the functional area (r>0.41)**	**Mean correlation outside the functional area**
**Tasks of interest**	**Hand motor**	9	0.498	0.107
	**Hand sensory**	4	0.515	0.131
	**Index motor**	3	0.520	0.132
	**Index sensory**	2	0.529	0.146
	**Thumb motor**	1	0.526	0.192
	**Thumb sensory**	2	0.483	0.119
	**Thumb/index motor**	3	0.494	0.111
	**Thumb/index sensory**	1	0.570	0.101
	**Ring finger/little finger sensory**	1	0.507	0.114
	**Wrist motor**	2	0.521	0.131
	**Wrist sensory**	1	0.472	0.186
	**Elbow/biceps sensory**	1	0.500	0.184
	**Mouth motor**	8	0.508	0.124
	**Mouth sensory**	3	0.505	0.121
	**Repetition (sentences)**	1	0.503	0.154
	**Count (1 to 10)**	1	0.461	0.073
	**Total**	**43**	**0.507**	**0.133**

Supplementary figure 1 - List of the different tested tasks depending on the lesion location. The number of times that one task was tested is indicated in the Number column. For every tested task, the mean correlation between the task pattern and the Doppler signal is documented along with the mean correlation calculated outside the functional area as a reference correlation value. In 43 cases, detection of the corresponding functional area (r>0.41) was obtained.

Supplementary video 1 - Neuroimaging of the human brain using intraoperative fUltrasound. First, this video presents the ultrasound probe positioning on the "mouth sensitive" cortical area (ESM tag 13). Second, the movie of the cerebral blood volume (CBV) variations compared to the baseline CBV is displayed in the imaging plane during 220 s of ultrasound acquisition. Stimulations ("MOUTH MOTION") result in a 20% increase in CBV compared to baseline CBV in the associated cortical area during stimulation. The drawing of the brain was created by Alexandre Dizeux.
